# Thalidomide in the treatment of human immunodeficiency virus-negative tuberculous meningitis: A case report

**DOI:** 10.1097/MD.0000000000022639

**Published:** 2020-10-02

**Authors:** Ping Liu, Ning Pei, Xuhui Liu, Wei Huang, Shuihua Lu

**Affiliations:** Department of Tuberculosis, Shanghai Public Health Clinical Center, Fudan University, Shanghai, China.

**Keywords:** case report, paradoxical tuberculosis, thalidomide, treatment, tuberculosis meningitis

## Abstract

**Introduction:**

Tuberculous meningitis (TBM) is the most fatal type of tuberculosis in which corticosteroids are added with antitubercular therapy to prevent permanent brain damage. However, this treatment may produce paradoxical reactions. In such cases, thalidomide use might reduce central nervous system inflammation and improve the outcome. We present the case of a human immunodeficiency virus-negative patient with TBM who developed paradoxical reactions manifesting as multiple intracranial tuberculomas that were resistant to standard care (antitubercular drugs and corticosteroids) but responded well to thalidomide.

**Patient's main concern and clinical findings:**

The patient was a 40-year-old Chinese female, who was admitted with a 10-day history of headaches, night sweats, and cough. She was healthy before contracting the infection and had no history of contact with tuberculosis patients.

**Diagnoses, intervention, and outcome:**

We diagnosed the patient with TBM complicated by the occurrence of pulmonary tuberculosis. Positive results were obtained from Gram and Ziehl-Neelsen staining of the sputum and acid-fast bacilli sputum culture. Standard treatment was initiated with antitubercular drugs (daily isoniazid, rifampicin, ethionamide, and pyrazinamide) and corticosteroids (dexamethasone). However, 3 months later the magnetic resonance imaging of the head revealed some new tuberculoma lesion. Thus, a specific therapy of antitubercular drugs and thalidomide was introduced. On completion of a 12-month course of antitubercular drugs with 2 months of thalidomide, the patient showed favorable outcomes without neurologic sequelae. Moreover, thalidomide appeared safe and well tolerated in the patient.

**Conclusion:**

In addition to the specific anti-tubercular and adjuvant corticosteroid therapies for TBM, thalidomide can be used as a “salvage” antitubercular drug in cases that are unresponsive to corticosteroids.

## 1 Introduction

Tuberculosis (TB) is a major global health problem. It is the ninth leading cause of death worldwide and is the cause of most deaths from a single infectious agent, ranking above human immunodeficiency virus (HIV) infections. In 2016,^[1]^ an estimated 10.4 million people fell ill because of TB. The most fatal type of TB is tuberculous meningitis (TBM) and is estimated to have more than 100,000 new cases per year. Moreover, a large study in Brazil^[2]^ reported that meningitis accounted for 6% of all extrapulmonary presentations in 57,217 patients with extrapulmonary TB. Approximately one-half of the patients with TBM either die or suffer severe neurologic disability. The standard protocol for management of such cases involves the administration of antitubercular drugs for 12 months or longer in conjunction with corticosteroids to reduce brain edema. However, in the course of this treatment, paradoxical reactions also known as the immune reconstructive inflammation syndrome (IRIS), may occur in patients.

TB-associated IRIS is defined as the worsening of a pre-existing lesion or the appearance of new lesion in a patient whose clinical symptoms initially improved with antitubercular treatment.[^3^,^4^,^5^] IRIS occurs in 2% to 14% of TB cases[^5^,^6^,^7^,^8^] and mostly manifests as an enlargement of a pre-existing lesion.^[6]^ It can occur in HIV-positive[^9^,^10^] and HIV-negative^[11]^ TB patients, and the phenomenon may be related to a sudden and violent immune reaction with a cytokine storm of tumor necrosis factor-alpha (TNF-α) and interferon-gamma.[^12^,^13^] In a rabbits model of TBM, increased local production of TNF-α within the exudate was closely related to pathogenesis.^[14]^

Thalidomide is a new immunomodulatory drug that is effective against cancer-associated inflammation, acute inflammation such as sepsis and pneumonia, and chronic infections that are related to the inflammatory state. Recently, extensive research on the pharmacological mechanism of thalidomide as a potent TNF-α inhibitor, has led to its use in TB, HIV, and other infections.

We describe the case of a patient with HIV-negative TBM who developed paradoxical reactions manifesting as multiple intracranial tuberculomas that were resistant to standard care (antitubercular drugs and corticosteroids) but responded well to thalidomide.

## 2 Patient information

The described case is of a 40-year-old female patient who presented with a 10-day history of headaches, night sweats, and cough. She was admitted to Shanghai Public Health Clinical Center in July 2017. The patient was previously healthy and had no other diseases.

## 3 Clinical findings

**Figure 1:**
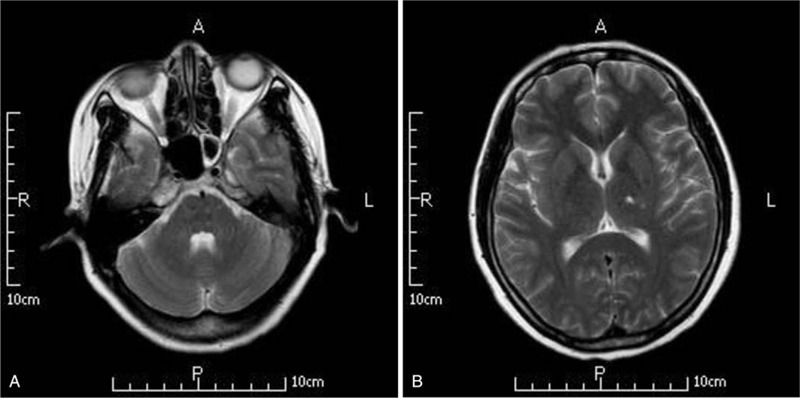
(A and B) Cerebrospinal fluid magnetic resonance imaging (MRI) of the brain showed in both sides of the cerebral cortex, the left basal ganglia, the left thalamus, the middle brain, the pontine, the right cerebellar hemisphere, there were many abnormal signals. T1WI showed low signal, T2WI, T2-FLAIR showed high signal, DWI showed high signal, the enhanced scan was ring strengthening, and no obvious abnormal enhancement was found in the meninges.

The patient was admitted with a 10-days history of headaches, night sweats, and little cough to our hospital, the patient had no obvious cause of headache more than 10 days ago, with bilateral forehead and top as the most serious. At that time, there was no fever, no obvious cough and chest tightness, no nausea and vomiting and night sweats. He had taken some medicine (unknown in details) and had no effect. She was treated in the local hospital. His Brain enhanced MR (Fig. 1) showed that there were multiple abnormal signals in bilateral cerebral hemispheres and brainstem, and metastasis was considered. The patient asked the superior hospital to continue diagnosis and treatment, so he was discharged from the local hospital and came to our hospital for outpatient treatment and treatment today.

## 4 Timeline

The patient had marked pulmonary TB and meningitis and was treated in the first month with antitubercular therapy consisting of daily isoniazid, rifampicin, ethionamide, pyrazinamide, and dexamethasone. The headaches, night sweats, and cough progressively disappeared within 2 weeks. Concomitantly, pulmonary tuberculosis was confirmed by the presence of Mycobacterium tuberculosis with full drug susceptibility (rifampicin, isoniazid, streptomycin, and ethambutol) in sputum cultures.

**Figure 2:**
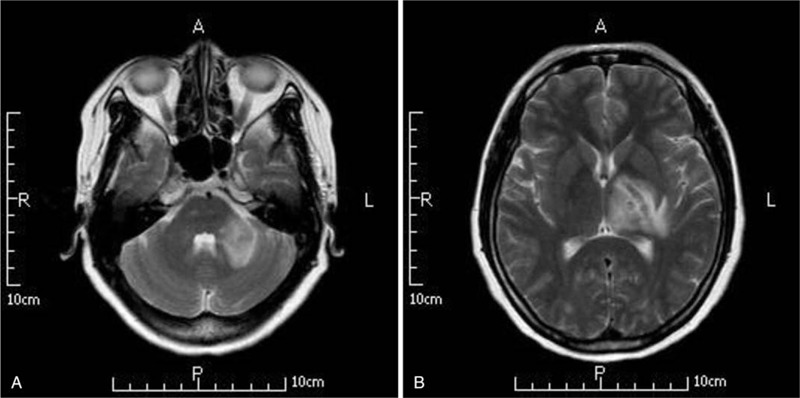
(A and B) The head MRI revealed some new lesion compatible with tuberculoma after a standard 3 mo care with anti-tuberculosis drugs and corticosteroids. MRI = magnetic resonance imaging.

Three months later, during a follow-up re-examination, magnetic resonance imaging (MRI) of the head revealed some new tuberculoma lesion (Fig. 2).

## 5 Diagnostic assessment

On investigation, the white blood cell count was 3.27 × 10^9^/L. Lumbar puncture revealed that the intracranial pressure was 175 mmH_2_O and cerebrospinal fluid (CSF) was clear, Pan test was negative. The glucose, protein, and chloride concentrations were 3.96 mmol/L, 400.0 mg/L, and 124.6 mmol/L, respectively. Results of the bacteriological cultures and herpes simplex virus polymerase chain reactions in the CSF were negative. The sputum was sent for Gram and Ziehl-Neelsen staining and acid-fast bacilli culture; the results were positive. Although the HIV-1 serology was negative, the cluster of differentiation (CD) count was low: CD4, 256 cell/uL; CD3, 414 cell/uL; CD8, 161 cell/uL; and CD45, 661 cell/uL.

MRI of the brain showed (Fig. 1) several abnormal findings in both sides of the cerebral cortex, left basal ganglia, left thalamus, middle brain, pontine, and right cerebellar hemisphere. T1 weighted image showed a low signal, while T2 weighted image, T2-weighted-fluid-attenuated-inversion-recovery, and diffusion-weighted imaging showed high signals. The scan showed ring-enhancing and no obvious abnormal enhancement was found in the meninges. Lung computed tomography showed a lesion in the middle lobe of the right lung and multiple miliary shadows in both lungs.

## 6 Therapeutic intervention

With the patient's consent, thalidomide was initiated as this immunosuppressive therapy has proven efficacious in corticosteroid-refractory IRIS. A dose of 200 mg of thalidomide was prescribed for a period of 2 months.

## 7 Follow-up and outcomes

**Figure 3:**
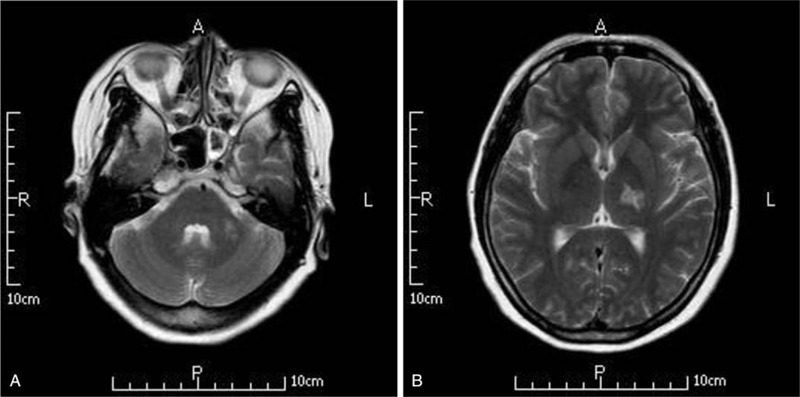
(A and B) Thalidomide treatment significantly improved the intracranial lesions after 2 mo.

At a follow-up examination after 2 months, the head MRI (Fig. 3) showed a regression of lesions. Subsequently, thalidomide therapy was stopped and no relapses were observed after 4 months of follow-up.

## 8 Discussion

TBM is the most devastating manifestation of a mycobacterium tuberculosis infection and represents a medical emergency. Anecdotal evidence suggests that corticosteroids reduce symptoms and inflammation in around 50% of patients with TBM. Therefore, national guidelines recommend adjunctive corticosteroids with antitubercular drugs to prevent death and disability from neurological TB. However, in some cases, corticosteroids have no effect and symptoms of TBM persist or worsen. Alternative anti-inflammatory agents have been tried in such circumstances, particularly in tuberculomas that involve the optic chiasm or in cases of optochiasmatic arachnoiditis that threaten the vision.[^13^,^15^,^16^]

Thalidomide, a drug that causes central inhibition, was found during the development of antibiotics. It is an anti-inflammatory, immunomodulatory, anti-angiogenic agent that is mainly prescribed as an anti-anxiety, hypnotic, antiemetic, and analgesic adjuvant. Thalidomide inhibits the TNF-α secretion of mononuclear cells and macrophages, and has strong anti-inflammatory properties.^[17]^ In a rabbit model of TBM, TNF levels correlate with the extent of pathology and treatment with antibiotics or thalidomide decreases TNF levels and protects against death.^[18]^ Therefore, this drug is currently used as an immunomodulator for the treatment of TBM, especially among patients with visual impairment and IRIS.[^13^,^15^,^16^] In a children's hospital in West Cape Town Province (South Africa), children with severe TBM were administered oral thalidomide in addition to antitubercular drugs and glucocorticoids; of the 15 children who participated in this study, 14 recovered and 1 died.^[19]^ The study showed that thalidomide significantly decreased TNF levels in CSF, and greatly improved the clinical manifestations and neuroimaging findings. Moreover, it showed that this drug is safe and well tolerated in patients with TBM. The anti-inflammatory effect of thalidomide may improve the blood supply of the brain, preventing the development of cerebral infarction and improving the functional recovery of the nervous system. Therefore, thalidomide can be used as a “salvage” antitubercular drug for TBM cases that are unresponsive to a large dose of glucocorticoids.

According to current specifications, the recommended dose of thalidomide starts from 6 mg/kg/d[^3^,^4^,^19^] for a period of 2 months. However, a dose of 12 to 24 mg/kg/d can be used as per the patient's condition and the course of the treatment can be extended to 8 months as per the duration of the disease.[^15^,^21^] Nevertheless, there are several side effects of thalidomide in addition to its historical teratogenicity. These include gastrointestinal reactions, hematological toxicity, cardiovascular toxicity, neurotoxicity, skin damage, pulmonary embolism, and others.^[20]^

In this study, we described a case of TBM with an intracranial tuberculoma that developed a paradoxical reaction to standard antitubercular care. On addition of thalidomide to the standard therapy, the treatment obtained favorable outcomes. Therefore, we conclude that thalidomide might be useful as a “salvage” antitubercular drug in similar cases. However, further research is warranted in the use of thalidomide as a treatment modality for TB and there is a need to evaluate the pros and cons of it in clinical application.

## 9 Patient perspective

The patient very grateful to the doctor for treating her disease, and she hope to share her successful experience with all of us.

## 10 Informed consent

This study was approved by the Ethics Committee of Shanghai Public Health Clinical Center, Fudan University, Shanghai, China. Informed written consent was obtained from the patient for publication of this case report and accompanying images.

## Author contributions

**Conceptualization:** Shuihua Lu.

**Data curation:** Ping Liu, Ning Pei, Xuhui Liu, Wei Huang.

**Formal analysis:** Ping Liu.

**Methodology:** Ping Liu, Ning Pei, Xuhui Liu, Wei Huang.

**Project administration:** Shuihua Lu, Ping Liu, Ning Pei, Xuhui Liu, Wei Huang.

**Supervision:** Shuihua Lu.

**Writing – original draft:** Ping Liu.

**Writing – review and editing:** Ping Liu, Ning Pei, Xuhui Liu, Wei Huang, Shuihua Lu.
